# Impact of Preoperative Handgrip Strength on Postoperative Outcome after Radical Gastrectomy for Gastric Cancer Patients

**DOI:** 10.3390/jcm11237129

**Published:** 2022-11-30

**Authors:** Ryota Matsui, Noriyuki Inaki, Toshikatsu Tsuji, Ryo Momosaki, Tetsu Fukunaga

**Affiliations:** 1 Department of Gastroenterological Surgery, Ishikawa Prefectural Central Hospital, Kanazawa 920-8530, Japan; 2 Department of Upper Gastrointestinal Surgery, Juntendo University Hospital, Tokyo 113-8431, Japan; 3 Department of Gastrointestinal Surgery/Breast Surgery, Graduate School of Medical Science, Kanazawa University, Ishikawa 920-8641, Japan; 4 Department of Rehabilitation Medicine, Mie University Graduate School of Medicine, Mie 514-8507, Japan

**Keywords:** gastric cancer, handgrip strength, muscle quality, muscle quantity, postoperative complication

## Abstract

In this study, we investigated whether preoperative low-handgrip strength (HGS) defined by the Asian working group for sarcopenia could be a predictor of postoperative outcomes in patients with gastric cancer. A total of 327 patients who underwent radical gastrectomy for c-stage I–III primary gastric cancer with pre-operative HGS records were included. The cut-off values of HGS were defined as 28 kg for males and 18 kg for females, with values below and above the cut-off defined as low-HGS and high-HGS, respectively. The primary outcome was infectious complications. We compared the postoperative outcomes of the groups after adjusting for the background using propensity score matching. Of the 327 patients, 246 (75.2%) and 81 (24.8%) were in the high and low-HGS groups, respectively. After adjusting for background, there were 57 patients in both groups. After matching, the low-HGS group had significantly more infectious complications (17.5% vs. 1.8%, *p* = 0.008). Multivariate analysis of infectious complications in the low-HGS group demonstrated chronic kidney disease and diabetes as independent risk factors (odds ratio 4.390, 95% confidence interval 1.120–17.20, *p* = 0.034). Preoperative low-HGS according to the Asian criteria was associated with infectious complications after gastrectomy. Chronic kidney disease and diabetes were independent risk factors for infectious complications among patients with low-HGS.

## 1. Introduction

In 2010, the European Working Group on Sarcopenia in Older People (EWGSOP) published a consensus definition of sarcopenia as a syndrome characterized by reduced skeletal muscle mass [1]. With the widespread use of preoperative body composition analysis, several studies have reported that decreased skeletal muscle mass appears to increase postoperative complications in patients with gastric cancer [2,3,4,5]. Although systematic reviews have shown that preoperative sarcopenia is an independent risk factor for postoperative complications in such patients [6,7,8], many studies have defined sarcopenia as reduced skeletal muscle mass. Low-hand grip strength (HGS), which is essential for a sarcopenia diagnosis, has not yet been fully investigated to determine whether it is involved in postoperative complications. EWGSOP revised its guidelines in 2018 and included reduced skeletal muscle quality in addition to reduced quantity to confirm a sarcopenia diagnosis [9]. Low muscle quality has also been reported to be a risk factor associated with postoperative complications [10,11,12,13,14]. Since HGS depends on muscle quantity and quality, it is important to determine whether complications can be predicted by HGS alone. Although the reported prevalence of preoperative sarcopenia in patients with gastric cancer varies widely from 7–70% [7], HGS in the diagnosis of sarcopenia is considered an important tool for predicting postoperative complications.

It has been reported that low-HGS is a predictor of postoperative complications [15,16]; however, the cut-off values for HGS have not been clarified. The Asian criteria for sarcopenia were revised in 2019 and the cut-off values for HGS have changed accordingly [17], but no study has investigated the validity of using these cut-off values to predict postoperative complications. Measuring HGS is a simple and inexpensive method that has long been used as an objective quantifier of muscle strength. HGS is assumed to decrease with age [9]; therefore, there is a need to develop and refine tools to assess HGS due to increasing rates of elderly patients.

We aimed to study the impact of preoperative HGS defined by the Asian Working Group for Sarcopenia on postoperative complications after radical gastrectomy for patients with gastric cancer. We hypothesized that low preoperative HGS increases postoperative complications in patients with gastric cancer.

## 2. Materials and Methods

### 2.1. Study Design

This retrospective study was conducted at the Ishikawa Prefectural Central Hospital and Juntendo University Urayasu Hospital. Patients who underwent radical gastrectomy for c-stage I–III primary gastric cancer who were diagnosed according to the 14th edition of the Japanese Classification of Gastric Carcinoma and had records of preoperative HGS between September 2014 and December 2020 were included. The inclusion criteria were as follows: (1) primary gastric cancer, (2) gastrectomy, and (3) preoperative HGS data. We excluded patients with (1) residual gastric cancer, (2) cancers of other organs, (3) non-gastrectomy surgical procedures, (4) unresectable distant metastases, (5) preoperative treatment, and (6) insufficient HGS data. We retrospectively collected clinical and laboratory data, including medical records and images, using the electronic patient record system of the hospital.

### 2.2. Data Collection

Preoperative HGS was measured twice in both hands with the patient in a standing position, and the mean was recorded [15]. Skeletal muscle mass index (SMI) was calculated by dividing the cross-sectional muscle mass area at the level of the third lumbar vertebrae by height in meters squared, which was measured using a computed tomography (CT) image one month before surgery. Visceral fat area (VFA) was likewise measured at the umbilical level. Skeletal muscle and visceral adipose tissue mass were analyzed using Ziostation (ZIOSOFT, Tokyo, Japan), with skeletal muscle tissue defined as within −29 to 150 HU and visceral adipose tissue as within −150 to −50 HU [18]. For muscle quality, CT values (HU) of regions of interest were measured at the umbilical level. We then calculated the intramuscular adipose tissue content (IMAC) by dividing the CT value of the multifidus muscles with that of subcutaneous fat, as presented in previous studies [19,20,21].

### 2.3. Cut-Off Values

The cut-off values of HGS were defined as 28 kg for males and 18 kg for females, as defined by Asian criteria [17], with values below and above the cut-off values defined as low-HGS and high-HGS, respectively. The cut-off values for IMAC and SMI were separately estimated using the median for males and females. The cut-off value for IMAC was −0.440 for males and −0.260 for females. Patients below the cut-off were categorized as low-IMAC, and those above the cut-off were categorized as high-IMAC. A higher IMAC indicated lower muscle quality. The cut-off value for SMI was calculated as 42.40 cm^2^/m^2^ for males and 33.18 cm^2^/m^2^ for females, while those below the cut-off were categorized as low-SMI, and those above the cut-off were categorized as high-SMI. Additionally, we also evaluated the previously reported cut-off values for HGS, SMI, and IMAC to diagnose sarcopenia in patients with gastric cancer. The cut-off values used in this study are shown in Table 1 [9,15,17,19,20,21,22,23,24,25,26].

### 2.4. Perioperative Management

Perioperative management was the same for all patients, with no preoperative carbohydrate loading, no perioperative artificial nutrition, no prehabilitation, and postoperative rehabilitation starting from postoperative day (POD) 1. All patients underwent mechanical bowel preparation the day before surgery and were administered epidural anesthesia. The nasogastric tube was immediately removed on the day of surgery. Oral intake was initiated on POD 2, beginning with water intake. The patients began to eat solid food on POD 3, starting with rice gruel, while they began to eat soft food on POD 4, and they advanced in three steps to regular food intake on POD 7. The patients were discharged when they had achieved adequate pain relief and soft food intake, returned to their preoperative mobility level, and had normal laboratory data on POD 7.

### 2.5. Outcomes

The primary outcome was infectious complications classified as Clavien–Dindo (CD) grade 2 or higher that occurred within 30 postoperative days. The secondary outcomes were total postoperative complications, severe postoperative complications as defined by Clavien–Dindo classification (CD) grade 3a or higher, operating time, intraoperative blood loss, length of postoperative hospital stay, and overall survival (OS). OS was defined as the period between surgery and death. For surgical site infections (SSIs), we collectively defined incisional SSIs as superficial, which included skin and subcutaneous tissue, and as deep, which included deeper soft tissue, such as fascia and muscle layers. Organ space SSIs were calculated as intra-abdominal abscesses. The high-HGS and low-HGS groups were compared in terms of postoperative outcomes after adjusting for background using propensity score matching (PSM), and multivariate analysis was performed using logistic regression analysis to identify factors related to infectious complications in the low-HGS group.

### 2.6. Clinicopathological Variables

The variables analyzed were sex, age, body mass index (BMI), surgical approach, surgical procedure, lymph node dissection, clinical stage, comorbidities, preoperative serum albumin (Alb), preoperative C-reactive protein (CRP), neutrophil-to-lymphocyte ratio (NLR), malnutrition defined by the global leadership initiative on malnutrition (GLIM), SMI, IMAC, and VFA. Chronic kidney disease (CKD) was defined as an estimated glomerular filtration rate (eGFR) < 60 mL/min/1.73 m^2^; diabetes was defined as either having a history of treatment or preoperative HbA1c ≥ 6.5%; chronic obstructive pulmonary disease was defined as forced expiratory volume in one second over forced vital capacity <70% on spirometry; and congestive heart failure was defined as either having a history of treatment or ejection fraction of less than 50% on echocardiography. NLR was calculated by dividing the total neutrophils by the total lymphocytes. We diagnosed GLIM-defined malnutrition in patients who were considered to be at risk in nutritional screening and had a positive phenotypic criterion and a positive etiologic criterion. The etiologic criterion was defined positive with decreased dietary intake or a CRP level of 0.5 mg/dL or higher before surgery. The phenotypic criterion was defined positive when the BWL rate was 5–10% within the past 6 months or 10–20% beyond 6 months, when BMI was <20.0 kg/m^2^ for patients <70 years old or <22.0 kg/m^2^ in patients ≥70 years old, or when SMI was below the cut-off value.

### 2.7. Statistical Analyses

To reduce the difference in patient background and selection bias in this non-randomized study, PSM was used for adjustment, and the patients were then divided into low-HGS and high-HGS groups. The propensity score was estimated using a logistic regression model with the following covariates: age, sex, BMI, clinical stage, surgical approach, surgical procedure, lymph node dissection, comorbidities, and VFA. The nearest neighbor matching method was applied, and one-to-one matching between the two groups was achieved. The caliper size was 0.20. After matching, the postoperative outcomes were compared between the two groups. Patient characteristics and postoperative outcomes were compared using the Mann–Whitney U-test for continuous variables and the chi-square test or Fisher’s exact test for categorical variables. In the low-HGS group, a multivariate analysis was performed for factors with *p*-values < 0.05, and a univariate analysis using logistic regression analysis was performed to identify factors related to infectious complications. For OS, the log-rank test was used for the Kaplan–Meier survival analyses. All statistical analyses were performed using EZR (Saitama Medical Center, Jichi Medical University, Saitama, Japan), which is based on R (The R Foundation for Statistical Computing, Vienna, Austria) and R commander. Statistical significance was set at *p* value < 0.05.

## 3. Results

### 3.1. Patient Characteristics

**Figure 1:**
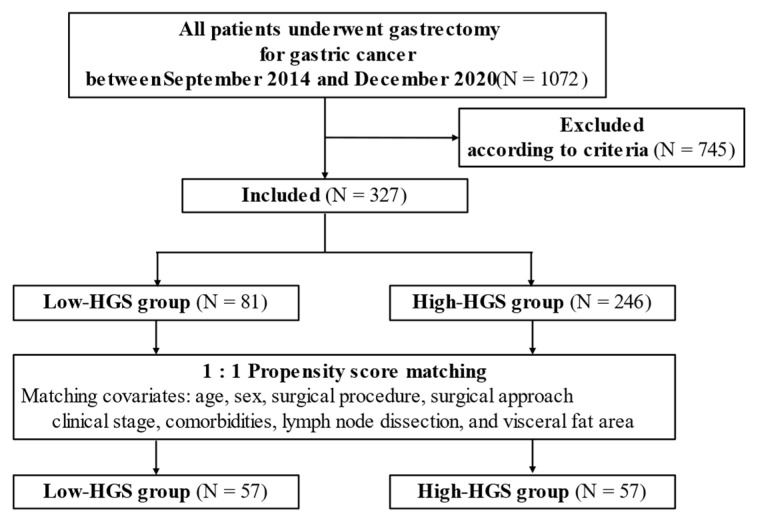
Study design.

The flowchart of this study is shown in Figure 1. In total, 327 eligible patients (68.16 ± 10.25 years, 235 males) were enrolled. The cut-off values for diagnosing sarcopenia and its prevalence are shown in Table 1. With cut-off values defined by Asian criteria, of the 327 patients, 246 (75.2%) were in the high-HGS group, and 81 (24.8%) were in the low-HGS group. Patient characteristics are shown in Table 2. The low-HGS group had significantly older individuals (*p* < 0.001), more females (*p* = 0.046), lower BMI (*p* = 0.006), more cases of clinical stage progression (*p* = 0.004), and more cases of GLIM-defined malnutrition (*p* < 0.001). In terms of body composition, the low-HGS group had a lower SMI (*p* <0.001) and a higher IMAC (*p* < 0.001). After adjusting for background, there were 57 patients in both groups, and there were no significant differences in any factors between the two groups.

### 3.2. Comparison of Postoperative Outcomes after Matching

Comparisons between the high-HGS and low-HGS groups for postoperative outcomes are shown in Table 3. There was no difference in the operating time, intraoperative blood loss, and length of postoperative hospital stay.

The low-HGS group had more postoperative infectious complications (*p* = 0.008). There were no differences in the total and severe complications. As for the breakdown of infectious complications, there were more superficial SSIs (*p* = 0.013), while pneumonia, intra-abdominal abscesses, anastomotic leakage, and pancreatic fistula were not different in the low-HGS group.

### 3.3. Independent Risk Factors of Infectious Complications in Low-HGS Patients

The results of the analyses regarding the risk factors of infectious complications in patients with low-HGS are shown in Table 4. In the univariate analysis, CKD (*p* = 0.012) and diabetes (*p* = 0.012) were significant. In the multivariate analysis, CKD (odds ratio [OR]: 4.390, 95% confidence interval [CI]: 1.120–17.20, *p* = 0.034) and diabetes (OR: 4.390, 95% CI: 1.120–17.20, *p* = 0.034) were significant independent risk factors.

### 3.4. Long-Term Prognosis Based on HGS

**Figure 2:**
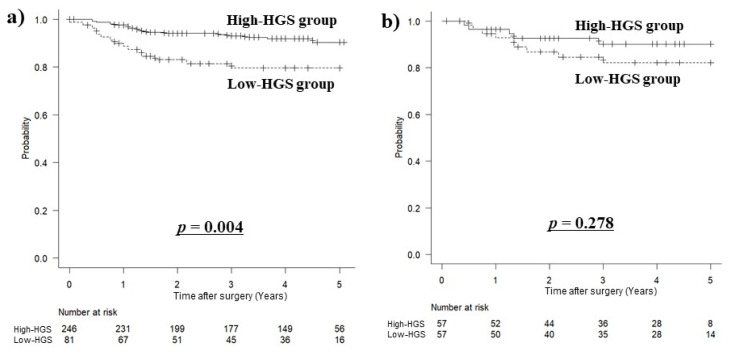
Kaplan–Meier survival curves for overall survival based on hand grip strength. (**a**) Before matching (*p* = 0.004) and (**b**) after matching (*p* = 0.278).

The median follow-up period was 48 months (interquartile range, 24–51 months). Before matching, the OS was significantly worse for the low-HGS group (hazard ratio (HR): 2.735, 95% CI: 1.389–5.386, *p* = 0.004) (Figure 2a). However, there was no difference in the OS after matching (HR: 1.831, 95% CI: 0.614–5.465, *p* = 0.278) (Figure 2b).

## 4. Discussion

This study is the first to show that low-HGS is a risk factor for infectious complications using the cut-off values defined by the Asian Working Group for Sarcopenia. After background adjustment for body composition in PSM, skeletal muscle mass, muscle quality, and visceral fat content did not differ between the two groups, suggesting that pure low-HGS may affect postoperative outcomes. The most common infectious complication was incisional SSI with a CD grade of 2 or higher. Multivariate analysis showed that CKD and diabetes were independent risk factors for infectious complications among patients with low-HGS. However, the long-term prognosis following gastrectomy was not affected when only hand grip strength differed, matching muscle quantity and quality.

Low-HGS was a risk factor for postoperative complications identified in many studies [2,3,4,5,15,16,27,28,29,30,31,32,33,34]. Many studies have used skeletal muscle mass and grip strength to diagnose sarcopenia; however, Wang et al. [4] compared patients with low-SMI who had high-HGS and those with low-HGS and found that major complications were significantly more frequent in the low-HGS group. This suggests that handgrip weakness is involved in complications, which is consistent with the results of this study. Additionally, Huang et al. [28]. pointed out that severe sarcopenia, in which low skeletal muscle mass, low-HGS, and gait speed decrease, increases the total number of postoperative complications, particularly pneumonia, compared with pre-sarcopenia, in which only skeletal muscle mass decreases. This result suggests that it is necessary to monitor whether muscle function is involved rather than skeletal muscle mass. These results suggest that an important factor is whether a decline in handgrip strength is due to low skeletal muscle mass or muscle quality, and the Asian criteria and its cut-off values may be a valid predictor of postoperative complications.

CKD and diabetes were risk factors for infectious complications in the low-HGS group. It has been previously reported that CKD is a risk factor for postoperative complications after gastrectomy, and the worse the eGFR, the higher the risk of complications [35,36,37]. In chronic renal failure, protein-energy malnutrition (PEM) is frequently observed, and the presence of metabolic acidosis is assumed to be associated with PEM by generating endocrine abnormalities, such as increased protein catabolism, decreased protein synthesis, and insulin resistance [38,39]. The low-HGS group had a low skeletal muscle mass, and the presence of PEM may have been involved. It has also been reported that diabetes is a risk factor for postoperative complications after gastrectomy [40,41,42,43]. Additionally, it has been reported that postoperative complications increase with multiple comorbidities [44], suggesting that although CKD and diabetes are independent risk factors, additional attention is warranted when these comorbidities coexist.

In the relationship between HGS and long-term prognosis, the overall cohort showed significantly poorer OS in the low-HGS group, especially in OCS. After matching, there was no statistically significant difference. This suggests that muscle mass, muscle quality, age, clinical stage, and comorbidities may affect HGS and have a greater impact on long-term prognosis than actual HGS. Reduced muscle quantity and quality have both been linked to poor long-term prognosis following gastrectomy [19,21,22,23,24,25,26]. As a result, the difference in HGS itself may suggest that it has little effect on long-term prognosis after gastrectomy.

Concerning the cut-off value for HGS, when the patients in this study were diagnosed with sarcopenia using each of the criteria in Table 1, even a small difference in the figure would affect the incidence of sarcopenia by several percent. In previous studies, cut-off values, such as the lower 20–25th percentile, have been established and discussed among the study participants; however, unless a cut-off value is established, the study cannot be developed as a validation study. Additionally, the diagnostic criteria for sarcopenia are anticipated to be revised in the future, and validation studies must be conducted to determine whether they are related to postoperative complications and long-term prognosis. In this study, we demonstrated that low-HGS according to current standards is a valid predictor of postoperative infectious complications.

The limitations of this study are as follows: (1) it was a two-center retrospective study, (2) it has a small sample size, and (3) the association between low-HGS and infectious complications is unknown. A large prospective multicenter study using the present cut-off values should be conducted to demonstrate the universality of the results of this study. In this study, there was no difference in skeletal muscle mass and muscle quality between the two groups after adjusting for background, suggesting that low-HGS itself may be involved in postoperative complications. Further research on the etiology of this disease is expected in the future. HGS is a valuable source of information to predict nutritional status, physical function, and outcomes, and its demand is anticipated to increase in the future. As a prospect, we would like to investigate whether prehabilitation and nutritional intervention improve postoperative outcomes in patients with preoperative low-HGS.

## 5. Conclusions

Preoperative low-HGS according to the Asian criteria was a factor associated with infectious complications after gastrectomy. Among patients with low-HGS, chronic kidney disease and diabetes were independent risk factors for infectious complications.

## Figures and Tables

**Table 1:** Cut-off values used in this study and their prevalence.

	Cut Off Values		Prevalence in This Study
	For Males	For Females	
Low-HGS: Asian [17]	28.0	18.0	24.8% (81/327)
European [9]	27.0	16.0	18.0% (59/327)
Sato et al. [15]	27.5	16.2	21.4% (70/327)
Median values	35.0	20.0	46.8% (153/327)
Low-SMI: Prado et al. [22]	52.4	38.5	78.9% (258/327)
Martin et al. [23]	53.0 for BMI ≥ 25	41.0	67.3% (220/327)
	43.0 for BMI ≤ 25		
Sakurai et al. [24]	43.2	34.6	51.7% (169/327)
Zhuang et al. [25]	40.8	34.9	41.0% (134/327)
Iritani et al. [26]	36.0	29.0	16.8% (55/327)
Median value	42.40	33.18	45.6% (149/327)
High-IMAC: Waki et al. [19]	−0.2541	−0.1095	13.5% (44/327)
Uchida et al. [20]	−0.430	−0.320	45.9% (150/327)
Watanabe et al. [21]	−0.245	−0.160	13.8% (45/327)
Median value	−0.440	−0.260	45.9% (150/327)

HGS: hand grip strength (kg); SMI: skeletal muscle mass index (cm^2^/m^2^); and IMAC: intramuscular adipose tissue content.

**Table 2:** Patient characteristics before and after propensity score matching.

	All Patients			After Matching		
	High-HGS (N = 246)	Low-HGS (N = 81)	*p* Value	High-HGS (N = 57)	Low-HGS (N = 57)	*p* Value
Sex Male	184 (74.8%)	51 (63.0%)	0.046	34 (59.6%)	33 (57.9%)	1
Female	62 (25.2%)	30 (37.0%)		23 (40.4%)	24 (42.1%)	
Age, mean ± SD	65.81 ± 10.08	75.30 ± 7.09	<0.001	73.81 ± 6.71	73.39 ± 6.43	0.733
Body mass index, mean ± SD	23.20 ± 3.24	22.04 ± 3.45	0.006	22.46 ± 3.22	22.56 ± 3.42	0.872
Surgical approach						
Laparoscopic surgery	214 (87.0%)	66 (81.5%)	0.272	45 (78.9%)	45 (78.9%)	1
Open surgery	32 (13.0%)	15 (18.5%)		12 (21.1%)	12 (21.1%)	
Surgical procedure						
Distal gastrectomy	178 (72.4%)	61 (75.3%)		43 (75.4%)	42 (73.7%)	
Proximal gastrectomy	16 (6.5%)	3 (3.7%)	0.723	4 (7.0%)	2 (3.5%)	0.621
Total gastrectomy	52 (21.1%)	17 (21.0%)		10 (17.5%)	13 (22.8%)	
Lymph node dissection	155 (63.3%)	57 (70.4%)	0.283	38 (66.7%)	41 (71.9%)	0.685
D1 + D2	90 (36.7%)	24 (29.6%)		19 (33.3%)	16 (28.1%)	
Clinical stage I	171 (69.5%)	42 (51.9%)		38 (66.7%)	33 (57.9%)	
II	31 (12.6%)	19 (23.5%)	0.004	6 (10.5%)	14 (24.6%)	0.124
III	44 (17.8%)	20 (24.6%)		13 (22.8%)	10 (17.6%)	
Comorbidity CKD	36 (14.6%)	17 (21.0%)	0.223	16 (28.1%)	12 (21.1%)	0.514
COPD	63 (25.6%)	13 (16.0%)	0.095	10 (17.5%)	9 (15.8%)	1
Diabetes	33 (13.4%)	17 (21.0%)	0.111	13 (22.8%)	12 (21.1%)	1
CHF	12 (4.9%)	7 (8.6%)	0.271	3 (5.3%)	2 (3.5%)	1
Albumin (g/dL), median (IQR)	4.30 (4.00–4.50)	3.90 (3.70–4.20)	<0.001	4.30 (3.80–4.40)	4.00 (3.70–4.30)	0.013
<3.0	3 (1.2%)	4 (4.9%)	0.071	3 (5.3%)	3 (5.3%)	1
CRP (mg/dL), median (IQR)	0.10 (0.10–0.20)	0.14 (0.10–0.30)	0.009	0.10 (0.10–0.30)	0.20 (0.10–0.40)	0.184
≥0.5	27 (11.0%)	14 (17.3%)	0.174	9 (15.8%)	10 (17.5%)	1
NLR, median (IQR)	2.24 (1.67–3.12)	2.09 (1.61–2.88)	0.48	2.05 (1.78–3.01)	2.09 (1.62–2.88)	0.773
GLIM-defined malnutrition	107 (43.5%)	63 (77.8%)	<0.001	35 (61.4%)	41 (71.9%)	0.321
SMI (cm^2^/m^2^), median (IQR)	41.41 (35.26–47.52)	36.12 (32.47–40.89)	<0.001	38.25 (31.26–44.70)	36.58 (33.13–41.05)	0.498
Low-SMI	96 (43.0%)	53 (70.7%)	<0.001	30 (52.6%)	37 (64.9%)	0.254
IMAC, median (IQR)	−0.43 (−0.51–−0.34)	−0.32 (−0.43–−0.22)	<0.001	−0.38 (−0.48–−0.25)	−0.36 (−0.44–−0.21)	0.372
High-IMAC	99 (44.2%)	51 (68.0%)	<0.001	30 (52.6%)	37 (64.9%)	0.254
Visceral fat area (cm^2^/m^2^),	81.20 (48.40–137.0)	72.30 (41.45–105.9)	0.081	81.20 (52.70–141.0)	74.90 (54.80–101.8)	0.216
Median (IQR)						

HGS: hand grip strength; SD: standard deviation; CKD: chronic kidney disease; COPD: chronic obstructive pulmonary disease; CHF: chronic heart failure; CRP: C-reactive protein; NLR: neutrophil-to-lymphocyte ratio; GLIM: global leadership initiative on malnutrition; SMI: skeletal muscle mass index; IQR: inter-quartile range; and IMAC: intramuscular adipose tissue content.

**Table 3:** Comparison of postoperative outcomes after matching.

	High-HGS (N = 57)	Low-HGS (N = 57)	*p* Value
Operating time (min), median (IQR)	208 (175–240)	205 (175–230)	0.536
Intraoperative blood loss (g), median (IQR)	12.0 (8.0–22.0)	14.0 (9.0–50.0)	0.359
Postoperative hospital stay (days), median (IQR)	12.0 (11.0–14.0)	13.0 (10.0–15.0)	0.547
Postoperative complications			
Infectious complications	1 (1.8%)	10 (17.5%)	0.008
Pneumonia	1 (1.8%)	1 (1.8%)	1
Incisional SSI	0 (0%)	7 (12.3%)	0.013
Intraabdominal abscess	0 (0%)	2 (3.5%)	0.496
Pancreatic fistula	0 (0%)	1 (1.8%)	1
Anastomotic leakage	0 (0%)	1 (1.8%)	1
Paralytic ileus	1 (1.8%)	1 (1.8%)	1
Severe complications	2 (3.5%)	1 (1.8%)	1
Total complications	7 (12.3%)	10 (17.5%)	0.6

HGS: hand grip strength; IQR: inter-quartile range; SSI: surgical site infection.

**Table 4:** Results of analyses of infectious complications in patients with low-HGS.

Variables	Univariate Analysis			Multivariate Analysis		
	OR	95% CI	*p* Value	OR	95% CI	*p* Value
Sex						
Female	1					
Male	3.41	0.695–16.80	0.131			
Age (years)						
<70	1					
≥70	4.18	0.505–34.60	0.185			
Surgical procedure Distal gastrectomy	1					
Total gastrectomy	1.66	0.441–6.230	0.455			
Surgical approach Laparoscopic surgery	1					
Open surgery	1.58	0.372–6.730	0.534			
Clinical stage						
I	1					
≥II	1.09	0.320–3.720	0.889			
Lymph nodes dissection						
D1+	1					
D2	0.182	0.022–1.500	0.113			
CKD						
Absent	1			1		
Present	5.27	1.430–19.40	0.012	4.39	1.120–17.20	0.034
Diabetes						
Absent	1			1		
Present	5.27	1.430–19.40	0.012	4.39	1.120–17.20	0.034
COPD						
Absent	1					
Present	1.05	0.203–5.490	0.95			
CHF						
Absent	1					
Present	0.955	0.105–8.720	0.967			
Albumin (g/dL)						
≥3.0	1					
<3.0	2	0.190–21.00	0.563			
CRP (mg/dL)						
<0.5	1					
≥0.5	1.76	0.409–7.550	0.448			
GLIM-defined malnutrition						
Absent	1					
Present	1.51	0.299–7.610	0.618			
SMI High-SMI	1					
Low-SMI Median value	2.33	0.466–11.60	0.304			
Lowest 25%	1.16	0.331–4.070	0.816			
Sakurai’s cut off	1.85	0.367–9.310	0.457			
Zhuang’s cut off	1.85	0.454–7.500	0.391			
Iritani’s cut off	1.93	0.540–6.920	0.311			
IMAC Low-IMAC	1					
High-IMAC Median value	2.68	0.539–13.30	0.228			
Highest 25%	1.33	0.378–4.690	0.656			
Waki’s cut off	0.902	0.218–3.730	0.887			
Uchida’s cut off	1.4	0.341–5.720	0.643			
Watanabe’s cut off	0.833	0.202–3.440	0.801			

OR: odds ratio; CI: confidence interval; CKD: chronic kidney disease; COPD: chronic obstructive pulmonary disease; CHF: chronic heart failure; GLIM: global leadership initiative on malnutrition; SMI: skeletal muscle mass index; and IMAC: intramuscular adipose tissue content.

## Data Availability

The datasets generated during and/or analyzed during the current study are available from the corresponding author on reasonable request.
